# Valuable insights from real-life experiences of advanced thyroid cancer treatment with sorafenib in Latin America

**DOI:** 10.20945/2359-3997000000398

**Published:** 2021-09-02

**Affiliations:** 1 Universidade de São Paulo Faculdade de Medicina Instituto do Câncer do Estado de São Paulo São Paulo SP Brasil Instituto do Câncer do Estado de São Paulo (ICESP), Faculdade de Medicina da Universidade de São Paulo (FMUSP), São Paulo, SP, Brasil

Approximately 10,000 new cases of thyroid cancer are diagnosed annually in Brazil (1), most of the cases are associated with a survival rate of over 98% 5 years after diagnosis, but 10-20% present or eventually develop distant metastases (2,3). Until recently, the only effective treatment for distant metastatic disease was radioactive iodine (RAI) and the prognosis of patients who failed RAI therapy was poor, the 10-yr survival rate being only 10% (4). From these, patients with enlarging and FDG-PET positive lesions are the ones with the lowest rates of survival (5). With the knowledge that oncogenic kinases play a significant role in tumorigenesis and disease progression several kinase inhibitors have been investigated and became approved therapies. Based on phase III, placebo-controlled trials, multi-kinase inhibitors such as sorafenib (DECISION) and Lenvatinib (SELECT) have been approved by the Federal and Drug Administration (FDA) in 2013 and 2015 and by ANVISA in 2015 and 2016, respectively.

In the DECISION trial (N=417), patients on sorafenib had significantly longer progression-free survival (PFS) compared to placebo (10.8 months versus 5.8 months (HR: 0.59; IC 95%: 0.45 – 0.76; p < 0.0001) as well as a higher response rate (RR) (12.2% versus 0.5%) with no difference in overall survival (OS). The most common adverse events, observed in more than 50% of patients, included hand and foot skin syndrome (HFS), diarrhea, alopecia, and rash. Hypertension was observed in 40.6% of patients (6).

In the SELECT trial (N=392), the median PFS was 18.3 months in those who received lenvatinib compared to 3.6 months in the placebo group (HR 0.21; IC 99%: 0,14 – 0.31; p < 0.001) and the RR was 64.8% versus 1.5% in the placebo arm. Taking into consideration the entire cohort there was no significant improvement in overall survival, but subgroup analysis identified improved OS in older patients (> 65 years) and in patients with lung metastases (> 1 cm) (7-9). Adverse events were frequent, 97.3% of patients experienced some form of an adverse event. Most frequently, patients experienced hypertension (67.8%), diarrhea (59.4%), fatigue (59%), weight loss among others (7).

New therapies for radioactive iodine refractory advanced thyroid cancer continue to emerge. Precision medicine has become a reality, mainly in private medicine, and the treatment is switching from promiscuous multi-targeted kinase inhibitors to the specific inhibition of the mutated pathway found in tumor genotyping. These more specific inhibitors associate significantly stronger inhibition with fewer adverse events. In this context, NTRK inhibitors such as Larotrectinib have been approved by Federal Agencies in the US, Europe, and Brazil; BRAF and MEK inhibitors such as dabrafenib with trametinib have FDA approval for anaplastic thyroid cancer that harbors a *BRAF* V600E mutation. Selpercatinib and Praseltinib were recently approved for *RET*-mediated cancers including medullary thyroid cancer and differentiated thyroid cancers (10-13).

In this issue of the *Archives of Endocrinology and Metabolism*, real-life experiences from two independent cancer centers in Latin America are published. The study by Fierro-Maya and cols. from Colombia reports the safety and efficacy of sorafenib in 19 patients with advanced differentiated thyroid cancer (DTC). This was a prospective, phase II study that included patients with iodine-refractory and progressive DTC. The primary outcome was RR by RECIST 1.1 criteria. Secondary outcomes included PFS, OS, duration of response, and safety. Eligible patients were initiated on sorafenib 400 mg twice a day and were followed 1 month after initiation of therapy and at 3-month intervals. Patients were allowed to have dose reductions according to the severity of adverse events. From 19 patients enrolled in the study, 6 were excluded from efficacy analysis since they did not complete 1 month of therapy. As expected, more than 80% of patients had papillary thyroid cancer (PTC), most had good performance status (ECOG 0 or 1), but 14 patients (73%) had a history of unspecified cardiovascular morbidity. From 13 evaluable responses, 5 had a partial response (PR) (35.7%) with a mean duration of 10.8 months, 6 patients had stable disease (SD) and 3 had progression of disease (PD). Median PFS and OS were not reached at the planned 2-yr follow-up period but were estimated as 18 months (mean, 95% CI 10.7-20.3) and 21.3 months (mean, 95% CI 17.8-24.8), respectively. In terms of significant AEs, HFS was observed in 68% of patients, diarrhea in 57%, hypertension in roughly 100% of patients. In addition, one patient had a myocardial infarction, and another patient suffered a sudden death possibly from a ruptured aortic aneurysm (14).

The study by Treitsman and cols. is a retrospective review of 44 patients with advanced DTC refractory to radioactive iodine therapy treated with sorafenib (N=40) or vandetanib (N=4) upon documented disease progression. In addition to analysis of RR, PFS and AEs, the authors compared tumor and clinical characteristics between patients with distinct outcomes related to disease progression and death, in an attempt to identify prognostic factors associated with good response to therapy. At this Institution, the indication to start MKI therapy included disease progression with symptoms, or asymptomatic with extensive disease. Patients that were asymptomatic with lesions < 2 cm were not started on therapy despite progression. Patients were followed by a multidisciplinary team at 15 or 30-day intervals for dose adjustments and to manage side effects. Similar to other studies, PTC was the most frequent histology, and lung was the most frequent site of metastases (91%), with 20% of patients having only lung metastases. Regarding the response to therapy, the authors observed very favorable responses, 1 patient had a complete response (CR), 9 PR (20.4%), 22 SD (50%), and 12 PD (27.3%), with a median PFS of 24 months and median OS of 31 months. Of 44 patients, 43 developed an AE, most were grade 1 or 2 (83%); half required drug interruption, and adjustment and 25% discontinued the drug due to an adverse event. The most frequent AEs were HFS (68.2%), diarrhea (70.4%), and fatigue (70.4%); hypertension was observed in 11.3%. According to univariate analysis, having a target lesion with high SUV in PET-CT, larger primary tumor size, and several metastatic sites were associated with poor response while having lung-only metastases and lower thyroglobulin levels during therapy were associated with better outcome (15).

Both studies provide valuable insights regarding the treatment of advanced thyroid cancer in Latin America where most patients depend on the public health system with limited resources, with sorafenib being the primary or only available drug. First, they demonstrate very favorable responses to therapy with patients benefiting from symptomatic improvement and prolonged PFS. In both studies, PFS was longer than observed in phase III DECISION trial but similar to other real-life experiences published to date (14). One possible explanation for this is the fact that in clinical practice, physicians might use less stringent criteria to define disease progression during treatment, especially given continued clinical benefit and lack of other therapeutic options. In regard to better response rate, one possible reason could be the predominance of lung metastases in both studies. Another important piece of information obtained from the INCA study (1) is the fact that initiating therapy at a later timepoint (target lesions > 2 cm) did not impact response rate and PFS. However, at the same time, it was clear that patients with more aggressive disease (higher SUV in PET-CT and extensive metastases) should not have initiation of treatment delayed. Despite these encouraging reports, one important message that should be drawn from both experiences is the rate of adverse events, therefore careful patient selection before initiation of therapy, better control of comorbidities (hypertension), and a close follow-up with frequent clinical visits performed by a multidisciplinary team is of utmost importance to avoid undesirable treatment discontinuation.
